# Microhardness and In Vitro Corrosion of Heat-Treated Mg–Y–Ag Biodegradable Alloy

**DOI:** 10.3390/ma10010055

**Published:** 2017-01-11

**Authors:** Marián Vlček, František Lukáč, Hana Kudrnová, Bohumil Smola, Ivana Stulíková, Monika Luczak, Gábor Szakács, Norbert Hort, Regine Willumeit-Römer

**Affiliations:** 1 Faculty of Mathematics and Physics, Charles University, Ke Karlovu 3, 12116 Prague 2, Czech Republic; Frantisek.Lukac@mff.cuni.cz (F.L.); Hana.Kudrnova@mff.cuni.cz (H.K.); smola@met.mff.cuni.cz (B.S.); stulik@karlov.mff.cuni.cz (I.S.); 2 Institute of Materials Research, Helmholtz-Zentrum Geesthacht, Max-Planck-Straße 1, 21502 Geesthacht, Germany; monika.luczak@hzg.de (M.L.); gabor.szakacs@hzg.de (G.S.); Norbert.Hort@hzg.de (N.H.); regine.willumeit@hzg.de (R.W.-R.)

**Keywords:** biodegradable implants, magnesium alloys, heat treatment, corrosion

## Abstract

Magnesium alloys are promising candidates for biodegradable medical implants which reduce the necessity of second surgery to remove the implants. Yttrium in solid solution is an attractive alloying element because it improves mechanical properties and exhibits suitable corrosion properties. Silver was shown to have an antibacterial effect and can also enhance the mechanical properties of magnesium alloys. Measurements of microhardness and electrical resistivity were used to study the response of Mg–4Y and Mg–4Y–1Ag alloys to isochronal or isothermal heat treatments. Hardening response and electrical resistivity annealing curves in these alloys were compared in order to investigate the effect of silver addition. Procedures for solid solution annealing and artificial aging of the Mg–4Y–1Ag alloy were developed. The corrosion rate of the as-cast and heat-treated Mg–4Y–1Ag alloy was measured by the mass loss method. It was found out that solid solution heat treatment, as well artificial aging to peak hardness, lead to substantial improvement in the corrosion properties of the Mg–4Y–1Ag alloy.

## 1. Introduction

Magnesium alloys, as possible biodegradable load-bearing implant materials, have intensively attracted the interest of researchers for more than one decade (e.g., [1,2,3]). Solutes used to increase mechanical properties can strengthen material via solution strengthening as long as the alloying elements remain in solid solution. Alloying elements in higher concentrations usually react with magnesium or among each other and develop intermetallic phases’ precipitates. Precipitation hardening depends not only on number density of precipitates but also on particle’s morphology and eventual orientation in the Mg-matrix [4]. Proper heat treatment procedures enabling dissolution and secondary phase precipitation have to be developed individually on a base of detailed investigation of the particular alloy [5]. Both solid solution strengthening and precipitation hardening improve strength, but deteriorate the alloy’s ductility. The magnesium alloys investigated as candidates for load-bearing implant materials are very often based on commercial alloys which have been developed for the transport and leisure industry [3,6,7]. Their complex compositions and microstructures enhance the number of variables and hence make an interpretation of in vitro and in vivo degradation studies difficult. More simple alloys are now preferred in the research community for in vitro experiments which could be used as a guidance for future alloy tailoring for Mg alloys production [8,9].

Yttrium appears to be one of the interesting alloying elements for degradable Mg implants as the same value of standard electrochemical potential of Y as of Mg (−2.37 V relative to the standard hydrogen electrode) promises particular corrosion properties of Mg–Y alloys [10]. Mg–Y-based alloys belong to the group of Mg alloys with better corrosion properties in NaCl solution [11]. Moreover, Y has a high maximum solubility in Mg and therefore a relatively high concentration of solid solution can be achieved. Corrosion in 0.1 M NaCl increases significantly with volume fraction of the equilibrium Mg_24_Y_5_ secondary phase due to micro-galvanic corrosion acceleration [12] while Y in solid solution can increase the protective nature of the surface film [3,13]. The beneficial role of Y on the corrosion of Mg alloy in cell culture medium was also reported [14]. Although some rare earth elements are toxic for the human body, Y has a relatively low toxicity and can be used in the medical treatment of patients [15,16]. The maximum solubility of Y in Mg is 12.5 wt % at 566 °C and decreases to 2.7 wt % at 200 °C. It promises the production of a wide range of solid solutions quenched from high temperatures as well as materials strengthened by the secondary phase. The equilibrium secondary phase β of Mg_24_Y_5_ composition has a cubic body centered structure (a = 1.13 nm) and forms as prismatic plates in the α-Mg matrix [5]. It is preceded by the development of β′ phase globular particles precipitating at lower annealing temperatures and having a base-centered orthorhombic lattice (a = 0.64 nm, b = 2.223 nm, c = 0.521 nm) [17]. The age-hardening response of solution treated Mg–Y alloys achieves almost 50% in alloys with the Y concentration above 8 wt %, although the aging at 200 °C is only several percent for lower Y concentrations [18].

Mg–Ag binary alloys with up to 6 wt % of Ag in Mg have been investigated in vitro and in vivo due to the well-known antibacterial effect of Ag [9,19,20]. Solid solution heat treatment of the alloys reduces the corrosion rate under cell culture conditions to one-third of that in the as-cast state. Corrosion properties deteriorate only a little after subsequent annealing to the peak hardness and the corrosion rate remains comparable to that of pure Mg [9,21]. In particular, the Mg–2 wt % Ag shows adjustable improved mechanical, corrosive, cytocompatible and antibacterial properties. The degradation rates in vitro and in vivo show no significant difference after 4 weeks testing time [9]. The equilibrium secondary phase in binary Mg–Ag alloys from the Mg rich corner is the Mg_4_Ag phase of the hexagonal structure (a = 1.2485 nm, c = 1.4412 nm) [22]. Even though the solubility of Ag in Mg (maximum 15 wt % at 465 °C) decreases steeply with decreasing temperature, the age hardening effect in binary alloys up to 6 wt % Ag (annealed at 440 °C for 16 h + 185 °C for 8 h) is only moderate [21]. Only few re-precipitates were observed by a scanning electron microscope in Mg–6 wt % Ag treated to peak hardening condition.

It is well established that an addition of proper minor elements to binary Mg alloys enhances significantly the number of fine precipitates developing during age hardening and increases mechanical properties considerably (e.g., Mg–4Y–1Mn–1Zn wt % [23], Mg–6Gd–0.6Zr wt % alloy with 1–2 wt % Zn [24], Mg–2.7Nd–0.6Zn–0.5Zr wt % [25], Mg–4Sm–1Zn–0.4Zr wt % [26], a wide range of Mg–Sn alloys with 0.1–0.5 wt % Zn [27] or Mg–4Zn–0.35Ca wt %). Among these micro additions, Ag enables relatively frequently a considerable improvement of age hardening. Peak hardness increases from 27% to 42% after alloying the Mg–6.2 wt % Zn by 0.4 wt % Ag. Number density of precipitates increases in one order of magnitude and the aspect ratio of precipitates is about 50% higher in the alloy containing Ag [28]. The exact role of Ag in the MgZn_2_ precipitation mechanism in early precipitation stages remains unclear while the concentration of Ag in the precipitates increases in the peak aged condition [29]. Systematic additions of Ag to the Mg–6Y–1Zn–0.6Zr (wt %) alloy promoted the formation of fine-scale precipitates, too. When the Ag concentration in the alloy was at and above 2 wt %, precipitation hardening was higher than 35%. This enhanced hardening response is associated with a dense distribution of fine-scale basal precipitate plates that were not observed in the Ag-free alloy [30]. The same addition of 2 wt % Ag in the Mg–6 wt % Gd increases hardness in the peak aged condition from 37% to 60%. This remarkable improvement in precipitation hardening is associated with the formation of a dense and uniform distribution of nano-scale basal precipitate plates within the matrix phase [31]. After all, the positive effect of Ag in Mg alloys is already expressed in commercial QE22 alloy with 2 wt % of Nd and 2 wt % of Ag. Peak hardening reaches almost 60% [32] and is most probably caused by oriented particles of two metastable phases [32,33], which is rarely observed in Mg alloys. It is generally accepted that Ag participates in the QE22 stable phase even though opinions about its composition developed from Mg_12_Nd_2_Ag with the complex hexagonal structure [34] to the (Mg,Ag)_12_Nd tetragonal phase [32,35,36].

The present work demonstrates thermal stability of mechanical properties and corrosion in a ternary Mg–4Y–1Ag alloy (nominal composition in wt %). Selection of alloying elements and their concentrations were governed by an attempt to keep the alloy reasonable for biomedical applications. The heat treatment parameters for solid solution annealing and the peak aged condition are introduced.

## 2. Experimental Details

Ingots with nominal compositions Mg–4 wt % Y and Mg–4 wt % Y–1 wt % Ag alloy were prepared by permanent mould direct-chill casting. For the casting, pure materials were used: Mg (99.95%), Y (99.95%) and Ag (99.99%). At first, the pure Mg was melted and heated up to 720 °C and then the preheated alloying elements were added during continous stirring. The melt was poured into a permanent steel mould (d = 110 mm, h = 220 mm) treated with boron nitride coating and placed in a holding furnace. After 10 minutes holding, the mould was immersed into running water with 1 mm/s speed [37]. During the full process, a protective atmosphere of Ar+2 wt %SF_6_ was used.

Chemical composition of the studied alloy determined by X-ray fluorescence (Y, Ag) is shown in Table 1.

Isothermal annealing as well as isochronal annealing with step 20 K/20 min were employed to study the response of the material to heat treatment. Isochronal annealing consists of a series of annealing steps which take the same time but the temperature for each subsequent step is increased by a fixed increment. For example, the 20 K/20 min regime used in this work implies that annealing steps were 20 min long and the temperature increment of 20 K was used. Each annealing step was finished by quenching.

Measurements of electrical resistivity were performed at 77 K by the DC four-point method with a dummy specimen in series. A constant direct current of 1 A was passed through samples during the measurement. Current reversal was used to suppress parasitic thermoelectromotive force and changes in electrical resistivity were measured with relative accuracy 10^−4^. H-shaped samples with dimensions approximately 70 mm × 10 mm × 2.5 mm for the electrical resistivity measurements were annealed in an oil bath and quenched into liquid nitrogen for annealing temperatures up to 240 °C. At an annealing temperatures higher than 240 °C, annealing in an Ar protective atmosphere and water quenching was used. Electrical resistivity changes measured at a low and stable temperature (e.g., in a liquid nitrogen bath) reflect changes in the phase composition of dilute alloys as the major contribution to the temperature-independent component of electrical resistivity comes from electron scattering by solutes in the matrix. Contributions of other lattice defects, such as point defects, dislocations or grain boundaries are usually one order of magnitude lower. The described electrical resistivity measurements were therefore used to monitor precipitation and dissolution processes in the studied alloys.

Vickers hardness (HV) was measured using microhardness tester Struers Duramin 300 (Struers ApS, Ballerup, Denmark), using the load of 100 g applied for 10 s. Samples for the microhardness measurements were annealed in air, quenched into water of room temperature and their surface was polished prior to the measurement. At least ten indentations were made into each sample.

The corrosion rate was determined by the mass loss method. Prior to the measurement, the surface of rectangular-shaped samples with dimensions 11 mm × 10 mm × 1.25 mm was prepared by grinding with sandpaper up to grit P2000. Samples of tested alloys were sterilized by sonication in ethanol and placed into a well plate. Each sample was immersed in 3 mL of high glucose Dulbeco’s Modified Eagle Medium (DMEM) with l-alanyl-l-glutamine (Thermo Fisher Scientific, Waltham, MA, USA) and sodium pyruvate + 10% Fetal Bovine Serum (FBS) (Biochrom GmbH, Berlin, Germany). A corrosion test was performed in the incubator under cell culture conditions (37 °C, 5% CO_2_, 19.7% O_2_, 95% relative humidity) and medium was changed every 2–3 days. After 168 h (7 days), samples were removed from the medium and washed in double distilled water. The corrosion products were subsequently removed by chromic acid. The immersion time of 168 h was selected to enable a comparison with already published data in similar alloys. A detailed investigation in [38] has shown that corrosion rates of solution treated binary Mg alloys with various rare earths determined by hydrogen evolution during immersion tests in 3% NaCl saturated with Mg(OH)_2_ accelerated to a steady state value after 3–7 days of immersion. Moreover, it was reported recently that, at this timepoint, the in vitro corrosion rate of commercialy-pure Mg is only slightly higher than the arterial in vivo corrosion rate and the difference is the smallest in the studied time range of approx. 14 days [39]. The corrosion rate was calculated from the mass difference Δ*m* between the initial weight of the sample and the sample mass after immersion and removal of corrosion products according to formula

*CR* (mm/year) = 8.76 × 10^4^ * Δ*m*/(*S* * *t* * *ρ*),

where *CR* is the corrosion rate in mm/year, *S* is the surface area of the sample in cm^2^, *t* is the immersion time in hours and *ρ* is the density of the sample in g/cm^3^. Six samples of each alloy at all three studied treatment conditions were measured to improve the statistical accuracy and to determine the uncertainty of the measurement.

Transmission electron microscopy (TEM) was carried out using a JEOL 2000FX microscope (JEOL Ltd., Tokyo, Japan). Samples of Mg–4Y alloy for examination in TEM were prepared by electrolytical polishing in Struers TenuPol-5 (Struers ApS, Ballerup, Denmark) at temperature −40 °C using electrolyte consisting of 20% HClO_4_ in ethanol. Specimens of Mg–4Y–1Ag alloy were thinned by electrolytical polishing in a solution of 5.3 g lithium chloride, 11.2 g magnesium perchlorate, 500 mL methanol and 100 mL 2-butoxyethanol at −40 °C and finished by ion beam polishing in Gatan Precision Ion Polishing System (Gatan, Munich, Germany).

**Figure 1:**
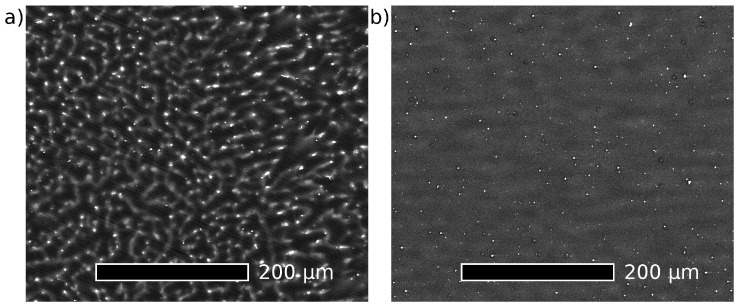
Scanning electron micrographs obtained in back scattered electron imaging mode of as-cast (**a**) Mg–4Y–1Ag alloy; (**b**) Mg–4Y alloy.

Scanning electron microscopy (SEM) performed in a Tescan MIRA microscope (Tescan, Brno, Czech Republic) was used to examine the microstructure of the Mg–4Y and Mg–4Y–1Ag alloys. Micrographs of polished samples obtained by detection of backscaterred electrons are shown in Figure 1. Homogenously distributed particles of secondary phases are present in both alloys in the as-cast condition. In addition, the presence of diffuse areas with a lighter shade of grey testifies the inhomogenous concentration of solutes in the Mg matrix. As can be seen in Figure 1, this inhomogeneity is significantly more pronounced in the Mg–4Y–1Ag alloy.

**Figure 2:**
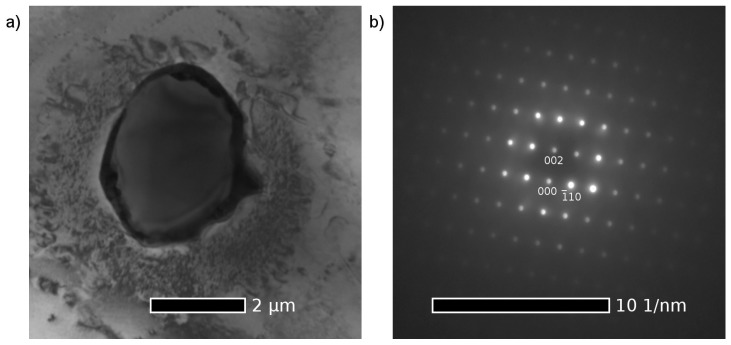
(**a**) Bright field TEM micrograph of the Mg_24_Y_5_ phase particle in the as-cast Mg–4Y–1Ag alloy with an Ag rich shell; (**b**) Corresponding selected area electron diffraction pattern of the particle in the [1,1,0] zone.

Since the cool down of the ingots to room temperature is relatively slow, it can be expected that particles of the equilibrium secondary phase β with the Mg_24_Y_5_ composition will be formed in Mg–Y alloys. Indeed, the structure of the secondary phase particles in the as-cast Mg–4Y–1Ag alloy was identified as Mg_24_Y_5_ (bcc structure, a = 1.13 nm); see Figure 2. Hence, the addition of 1 wt %Ag in the Mg–4Y–1Ag does not change the type of the stable phase and the precipitation sequence in the Mg–4Y–1Ag alloy ends with the same equilibrium phase as in binary Mg–Y alloys.

## 3. Results and Discussion

**Figure 3:**
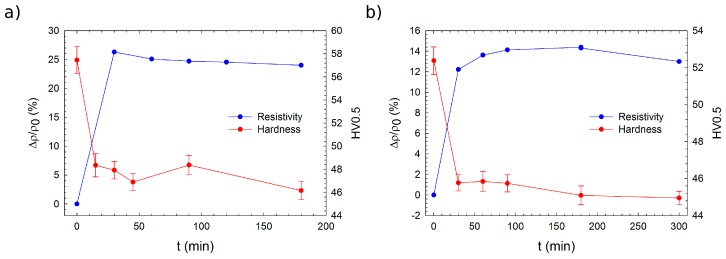
Development of relative resistivity changes measured at 77 K and of Vickers microhardness measured at room temperature due to solution treatment of (**a**) Mg–4Y–1Ag at 525 °C; (**b**) Mg–4Y alloy at 500 °C.

**Figure 4:**
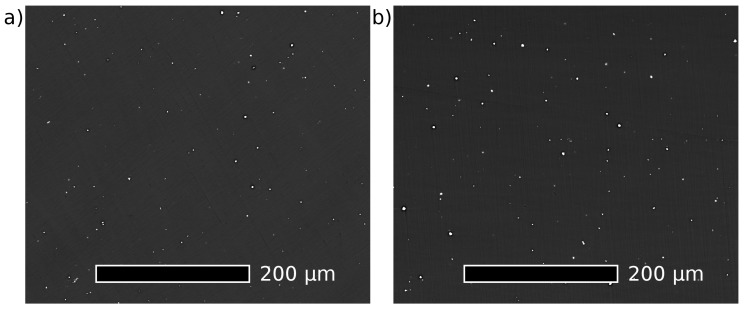
Scanning electron micrographs obtained in the back scattered electron imaging mode of solution treated alloys: (**a**) Mg–4Y–1Ag at 525 °C for 3 h; (**b**) Mg–4Y alloy at 500 °C for 5 h.

**Figure 5:**
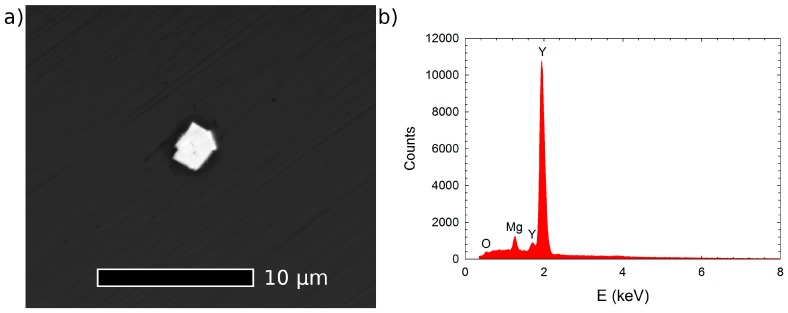
(**a**) Scanning electron micrograph of a rectangular particle in Mg–4Y–1Ag alloy solution treated at 525 °C for 3 h obtained in back scattered electron imaging mode; (**b**) Energy-dispersive X-ray spectrum acquired from the bright particle shown in (**a**) at 10 kV.

The as-cast Mg–4Y–1Ag alloy was solid solution heat treated at 525 °C analogically as the commercial QE22 (Mg–Ag–Nd) alloy [40]. Phase diagrams of the binary Mg–Y system indicate that a temperature of 500 °C is sufficient for achieving a solid solution in the Mg–4Y alloy [41,42]. Hence, this slightly lower temperature was used to solution-treat the as-cast Mg–4Y alloy in order to suppress the formation of hydrides [43,44]. Figure 3 shows relative resistivity changes in response to isothermal annealing at temperatures selected for solution treatment. Electrical resistivity of both alloys increases considerably due to a dissolution of secondary phases and already reaches maxima after relatively short annealing times (0.5 h and 1 h for the Mg–4Y–1Ag and the Mg–4Y respectively). No significant resistivity changes were observed in the Mg–4Y–1Ag alloy in the course of the prolonged heat treatment up to 3 h as well as in the binary alloy due to heating up to 5 h. SEM revealed that solutes segregation disappeared in both alloys after the high temperature annealing finished by quenching (525 °C/3 h for the Mg–4Y–1Ag alloy and 500 °C/5 h for the Mg–4Y alloy); see micrographs presented in Figure 4. Homogenous supersaturated solid solution developed and only few rectangular particles (1 μm order of magnitude large) with a very high content of Y (>80 wt %) can be observed in SEM after the solution treatment (T4 condition). Details of such a particle in the solution-treated Mg–4Y–1Ag alloy, together with its energy dispersive X-ray spectrum, are shown in Figure 5. These particles are most probably yttrium hydrides [43,44] formed either during the casting procedure or during the solid solution treatment. The dissolution of secondary phases into solid solution is connected with Vickers hardness decrease, reaching ~20% in both investigated alloys as the solid solution strengthening is weaker than the dispersion or precipitation hardening due to the secondary phase particles introduced during casting.

**Figure 6:**
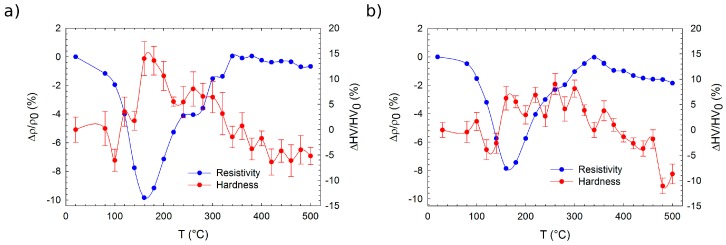
Isochronal annealing curves of relative resistivity and microhardness changes of solution treated alloys: (**a**) Mg–4Y–1Ag; (**b**) Mg–4Y. *ρ*_0_ and HV_0_ are values of electrical resistivity and Vickers microhardness in the solution-treated condition. The sample of the Mg–4Y–1Ag alloy was solution-treated at 525 °C for 3 h, while the sample of the Mg–4Y–1 alloy was subjected to solution treatment at 500 °C for 5 h.

The electrical resistivity response of the Mg–4Y–1Ag alloy solution treated at 525 °C for 3 h to isochronal step-by-step annealing up to 500 °C shows a decrease, already starting at low annealing temperatures and followed by an increase after annealing at temperatures higher than 160 °C—Figure 6a. The increase is slowed down in the annealing temperature range 240 °C–280 °C. Resistivity reaches almost the initial value after annealing at 340 °C and changes insignificantly on further annealing. The resistivity annealing curve indicates that the main precipitation process can be expected at temperatures lower than 160 °C and another phase transformation proceeds probably due to annealing in the range 240 °C–280 °C. The annealing resistivity curve of the Mg–4Y alloy solution treated at 500 °C for 5 h has the same character as that of the Mg–4Y–1Ag alloy—Figure 6b. Maximum solubility of Y in Mg at various temperatures which is ~2 wt % at 200 °C and ~4 wt % at 300 °C [45] and actual concentration in the studied alloys can explain an absence of pronounced resistivity changes after annealing at ~340 °C and higher temperatures. A slight resistivity decrease on annealing above 340 °C in both alloys can reflect the formation of yttrium hydrides [44]. The absolute resistivity decrease during the precipitation process is less pronounced in the Mg–4Y alloy than in the Mg–4Y–1Ag alloy. This can be expected due to a somewhat higher solute concentration in the Mg–4Y–1Ag alloy and is therefore due to a higher matrix supersaturation of the initial state. It agrees also with the fact that precipitation hardening is more pronounced in the material with Ag (~15%) than in the Mg–4Y alloy (~8%)—Figure 6. Temperature position of peak hardening agrees very well with the minimum of electrical resistivity in the Mg–4Y–1Ag alloy (Figure 6a) whereas microhardness of the Mg–4Y alloy remains almost constant over an extended range of annealing temperatures (Figure 6b). A poor microhardness response of the studied alloy to isochronal annealing coincides with the results of thermal stability investigation in a more concentrated binary Mg–Y alloy [18]. A beneficial effect of Ag on precipitates’ number density, already observed in some binary and more complex Mg alloys [28,29,30,31], may also not be excluded here. Since no precipitates were observed by TEM in the Mg–4Y alloy isochronally annealed up to 160 °C, which corresponds to the minimum of electrical resistivity (Figure 6b), one can conclude that this minimum relates to a development of solute clusters. This is in agreement with results of High-Angle Annular Detector Dark-Field Scanning Transmission Electron Microscopy (HAADF-STEM) studies reported in literature, where solute clusters were observed in a more concentrated Mg–Y alloy after long annealing at 150 °C [46]. At temperatures above 180 °C, these clusters partially dissolve as is testified by an increase in electrical resistivity. Since the coarse intermetallic particles present in the as-cast alloys were identified by TEM, such as Mg_24_Y_5,_ one can expect that the precipitation sequence will continue with the development of the metastable β′ phase with a base-centered orthorhombic lattice (a = 0.64 nm, b = 2.223 nm, c = 0.521 nm) [17] and finally ending with the precipitation of the equilibrium β phase with Mg_24_Y_5_ composition and a cubic body centered structure (a = 1.13 nm) [5]. Small undulations in the curves of electrical resistivity shown in Figure 6 between 180 °C and 340 °C might be caused by precipitation of these phases.

**Figure 7:**
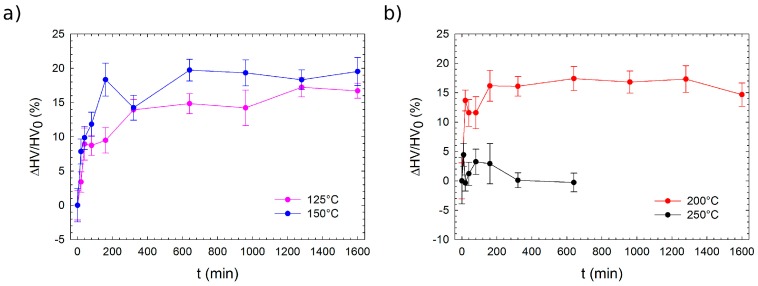
Response of microhardness to artificial aging of the solution-treated Mg–4Y–1Ag alloy at temperatures: (**a**) 125 °C and 150 °C; (**b**) 200 °C and 250 °C. Samples of the Mg–4Y–1Ag alloy were solution treated at 525 °C for 3 h prior to artificial aging.

In order to exploit the potential of the investigated ternary alloy, isothermal heat treatment was performed at various temperatures and times up to 26 h. Precipitation hardening was studied by means of microhardness measurements. Figure 7a,b shows isothermal annealing curves in the temperature range of 125 °C–250 °C. Although the heating at 250 °C, leading to very fast over aging and precipitation hardening, was negligible, annealing at lower temperatures causes a relatively useful hardening near 20%. The microstructure of the Mg–4Y–1Ag alloy aged at 150 °C for 16 h was investigated by TEM. However, no precipitates were observed and the obtained diffraction patterns consisted solely of reflections belonging to the Mg matrix and the MgO on the surface of the TEM foil. It can be therefore assumed that the peak hardening corresponds to a development of small solute clusters which are not observable in conventional TEM. Well-developed solute clusters were observed in a more concentrated Mg–Y alloy by HAADF-STEM until after annealing for 720 h at 150 °C [46]. The annealing time of 16 h at 150 °C, which was used to achieve the T6 condition for the Mg–4Y–1Ag, is much shorter. Therefore, the development of solute clusters will be most likely in its early stage only, but extremely long-time annealing for the T6 condition is technically not reasonable. It was experimentally proven that even 60 days at 150 °C were not enough to reach a maximum hardness increase in a solution-treated Mg–6 wt % Y alloy [47]. Thermal stability of hardness in the T6 state is acceptable and 10 h at 150 °C is sufficient to reach this condition.

**Figure 8:**
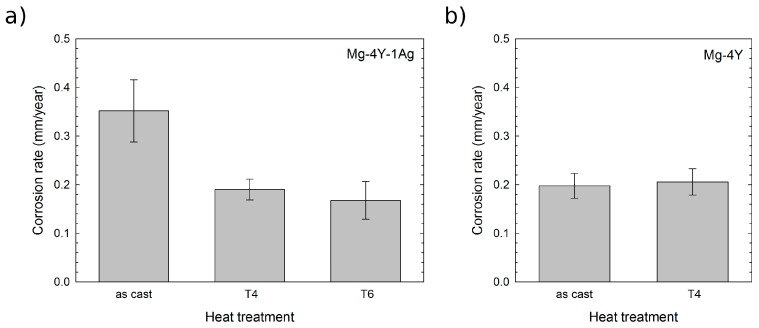
The corrosion rate of as-cast and heat-treated alloys: (**a**) Mg–4Y–1Ag; (**b**) Mg–4Y. For the Mg–4Y–1Ag alloy, heat treatment 525 °C/3 h followed by water quench was chosen for the T4. The same treatment, followed by aging 150 °C/16 h and water quenching, was used in the T6 condition. For the Mg–4Y alloy, T4 heat treatment was performed at 500 °C/5 h, followed by water quench.

**Figure 9:**
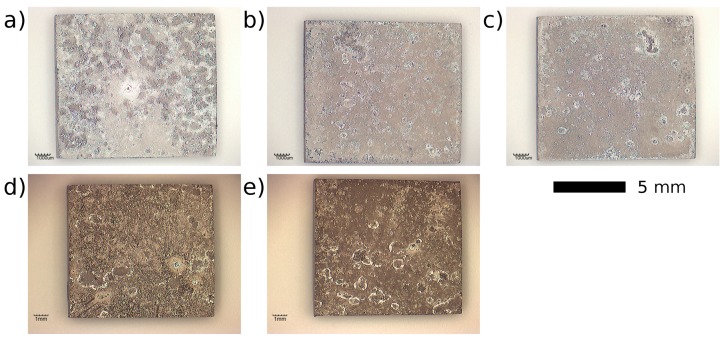
Images of as-cast and heat-treated samples of studied alloys after the corrosion test: (**a**) Mg–4Y–1Ag as-cast; (**b**) Mg–4Y–1Ag T4; (**c**) Mg–4Y–1Ag T6; (**d**) Mg–4Y as-cast; (**e**) Mg–4Y T4. See Figure 8 for a description of the heat treatment parameters.

The corrosion rate of the studied alloys in as-cast and heat-treated conditions determined by the mass loss method is shown in Figure 8. The corrosion rate of the Mg–4Y–1Ag is significantly reduced (to almost one half of the value of the as-cast state) by solution treatment while artificial aging has only a negligible effect. Dissolution of coarse Mg_24_Y_5_ particles and homogenization of solutes in the as-cast alloy matrix decrease the corrosion rate almost to one half of the original value. This is in agreement with measurements which show that the presence of the equilibrium Mg_24_Y_5_ secondary phase accelerates the degradation by micro-galvanic corrosion [12]. On the other hand, the fine precipitates formed during artificial aging have virtually no effect on the corrosion rate within experimental accuracy. Unlike the Mg–4Y–1Ag alloy, the corrosion rate of the Mg–4Y alloy was virtually unaffected by the solution treatment. This difference is most likely caused by better homogeneity of the Mg–4Y alloy in the as-cast condition, see Figure 1. The measured corrosion rates of the solution treated (T4) Mg–4Y–1Ag and Mg–4Y alloys are practically identical within experimental accuracy. Images of selected as-cast and heat-treated samples of the studied alloys after the mass loss corrosion test are presented in Figure 9. In addition to general corrosion of the samples, localized corrosion in the form of wide shallow pits is also present on all the studied samples. However, pitting is more pronounced in the as-cast materials when compared to the heat-treated samples due to a better homogeneity of alloying elements achieved by the solution treatment; compare Figure 1 and Figure 4. To obtain the data necessary for a discussion about a possible corrosion mechanism, time development of the corrosion rate would be required. The performed tests show that the addition of silver in the studied Mg–4Y–1Ag alloy does not deteriorate corrosion properties in both T4 and T6 conditions when compared to the binary Mg–4Y alloy.

Measured values of the corrosion rate indicate that the Mg–4Y–1Ag alloy is relatively slowly corroding the alloy among magnesium alloys investigated for biomedical applications [48,49]. This is advantageous since too fast corrosion can lead to the formation of hydrogen filled cavities and adverse pH changes in the vicinity of an implant [50]. Furthermore, mechanical stability of an implant has to be maintained for approximately 4 months for biomedical stents [51] and for 3–6 months for fracture fixation [52]. However, it has to be noted that there is some difficulty in estimating the corrosion rate in vivo from in vitro data [49]. In general, the in vitro corrosion rates tend to be faster than the corrosion rate measured in vivo [49]. Results obtained for pure Mg suggest that the corrosion rate in vivo is 1.2–1.9 ± 0.2 times faster [39]. In another study, multiplier 2.3 ± 0.2 was obtained when mechanical behavior and time to failure of pure Mg implants in an artery were considered [53]. Although these works showed substantial progress in establishing an in vivo–in vitro correlation, an in vivo study would still be required to fully assess the corrosion behavior and biological response of the body to the Mg–4Y–1Ag alloy.

## 4. Conclusions

Heat treatment parameters for solution treatment and artificial aging of the Mg–4Y–1Ag alloy were determined by microhardness tests and measurements of electrical resistivity. The addition of silver in the Mg–4Y–1Ag alloy enhances the hardening response. The corrosion rate of the Mg–4Y–1Ag alloy can be substantially decreased by solid solution annealing which dissolves intermetallic phases present in the as-cast condition and homogenizes the distribution of solutes in the magnesium matrix. Fine precipitates formed during artificial aging of the Mg–4Y–1Ag alloy do not seem to have a detrimental effect on the corrosion rate. The addition of 1 wt % Ag to the Mg–4Y alloy has practically no effect on the in vitro corrosion rate in the solution-treated condition.

## Figures and Tables

**Table 1:** Composition of studied alloys.

Alloy	Y (wt %)	Ag (wt %)
Mg–4Y	3.45	–
Mg–4Y–1Ag	4.05	1.12
